# Hydroxide and Hydrophobic Tetrabutylammonium Ions at the Hydrophobe–Water Interface

**DOI:** 10.3390/molecules30040785

**Published:** 2025-02-08

**Authors:** Alex M. Djerdjev, James K. Beattie

**Affiliations:** School of Chemistry, University of Sydney, Sydney, NSW 2006, Australia

**Keywords:** dielectric decrement, hydroxide ions, tetrabutylammonium, oil–water interface, pristine oil–water emulsion

## Abstract

Water and oil do not mix. This essential statement of the hydrophobic effect explains why oil-in-water (O/W) emulsions are unstable and why energy must be supplied to form such emulsions. Breaking O/W emulsions is an exothermic event. Yet metastable O/W emulsions can be prepared with only water acting as the stabilizer by the adsorption of hydroxide ions formed from the enhanced autolysis of interfacial water. The heat of desorption of the hydroxide ions from the oil–water interface is not directly accessible but is obtained from the difference between the heat of reaction and the sum of the neutralization and interfacial heats when an emulsion is broken by the addition of acid. This experimental value of 28.4 k_B_T is in good agreement with the theoretical estimate of 16–20 k_B_T made from the fluctuation/correlation model of the hydrophobic force and the value of 14 k_B_T obtained recently from surface spectroscopy. Subsequent verification of the force driving ions to hydrophobic surfaces is shown for tetrabutylammonium bromide with a dielectric decrement value of 26 M^−1^ compared to 20 M^−1^ for NaOH. The positive cation preferentially adsorbs at the oil–water interface over hydroxide ions in agreement with the predicted model.

## 1. Introduction

### 1.1. Hydrophobic Water Interfaces

That water and oil do not mix is an essential statement of the hydrophobic effect and explains why oil-in-water (O/W) emulsions are unstable and why energy must be supplied to form such emulsions. However, metastable O/W emulsions can be prepared with only water acting as a stabilizer. The earliest works on surfactant free interfaces began over 150 years ago by Quincke, who noted that air bubbles must be negatively charged, since they migrated towards the positive electrode when measured by electrophoresis [1]. Since then, it has been known and accepted that hydrophobic particles acquire a negative charge when suspended in neutral water [2,3]. Evidence of this effect has been observed experimentally on oil–water interfaces, solid hydrophobic surfaces, such as Teflon, and on gas bubbles [4,5,6,7].

**Figure 1:**
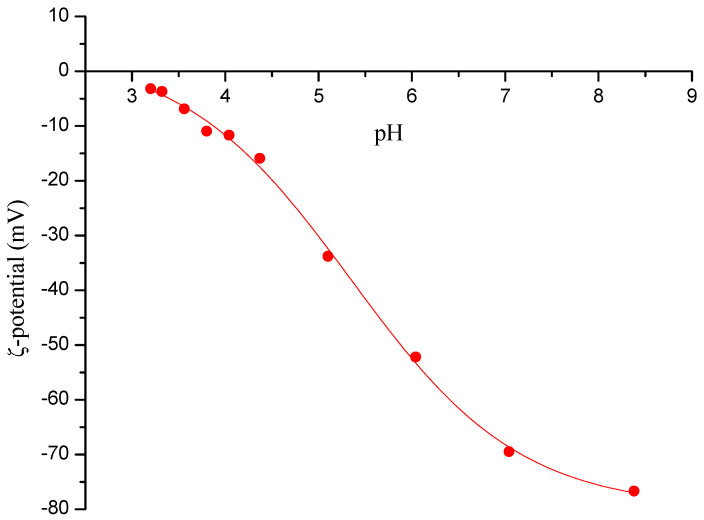
pH dependence of zeta potential of hexadecane drops in 0.2 mM NaCl.

Dickinson [8] proposed two possible mechanisms for hydroxide ion adsorption at hydrophobic interfaces. Firstly, hydroxide ion adsorption depends on the pH and saturates at pH 9 due to saturation of the interfacial surface. Secondly, adsorption of a molecular layer of water occurs at the hydrophobic interface where some of the interfacial water ionizes and partitions hydronium ions towards the aqueous bulk and hydroxide ions towards the interface. At higher pH, ionization would increase giving rise to a larger charge. Titration of the surface hydroxide with acid and measurement of the zeta potential displays the characteristic sigmoidal curve, with an inflexion point at pH 5.5, to support both mechanisms (Figure 1). Dukhin [9] recently demonstrated that the long-term stability of nanobubbles could be explained by the generation of an electric double layer at the interface caused by the adsorption of OH^−^ ions, which orients water molecules in the first layer. This electric field exerts a force on the water molecules that opposes the Laplace pressure almost exactly, but in the opposite direction. The stable bubble size depended on the hydroxide potential at the air–water interface. Xiong [10] demonstrated by stimulated Raman excited fluorescence microscopy, that a strong electric field at the oil–water interface of microdroplets aligns water dipoles with respect to the interface where the electric field is likely to arise from charge separation caused by the adsorption of negative ions at the water–oil interface.

Many techniques, such as spectroscopy, surface charge and surface tension measurements, as well as simulation methods, have been used to argue for the mechanism of charge generation at hydrophobic–water interfaces. Many studies support hydroxide ion adsorption [11,12,13,14,15,16,17], hydronium ions [18,19,20,21], or both [22,23,24]. Recent spectroscopic studies have shown an affinity for hydroxide over hydronium ions [17,25,26]. Tian and Shen, using phase-sensitive sum frequency vibrational spectroscopy, showed that the OH^−^ has the highest adsorption energy compared to those of H_3_O^+^ and Cl^−^ at a water–silane interface [26]. A study with second harmonic generation spectroscopy showed that hydroxide ions exhibit an affinity for the water–oil interface [17]. However, Iyota [14], using a modified Poisson–Boltzmann equation, showed that, from the concentration dependence of the surface density, not only OH^−^ ions, but also Cl^−^ ions and HCO_3_^−^ and/or CO_3_^2−^ ions, adsorb specifically at the water–air interface.

Recently, other mechanisms have been proposed involving charge–transfer processes [27,28,29]. Poli [27], using state-of-the-art linear scaling density functional theory (LS–DFT)-based simulations, provided evidence that charge transfer along hydrogen bonds and asymmetries in the hydrogen bond network due to topological defects can lead to the accumulation of negative surface charge at both oil–water and air–water interfaces. Pullanchery [29] showed that, from the spectral shifts of interfacial O–D and C–H modes, the structure of water in contact with hydrophobic droplets dispersed in water was characterized by charge–transfer interactions between water and oil molecules. Water at the oil droplet surface had a stronger H-bonding network compared with the planar air–water interface. Charge transfer from water to oil explained the negative charge and stability of bare oil droplets in water.

Indeed, the charging mechanism has been the topic of hot debate of late. An excellent recent review by Perrin [30] outlines the many proposed theories and observations on the charge at hydrophobic–water interfaces. However, no theory exists that can explain the formation of both negative and positive charges at hydrophobic–water interfaces. Charge transfer mechanisms do not account for the adsorption of hydrophobic ions, such as tetra-butyl ammonium ions, and hydronium ion adsorption does not generate positive surfaces at near neutral pH. We demonstrate that both hydroxide ion and tetra-butyl ammonium ions are attracted to the hydrophobic–water interface, which is consistent with the Hamaker-like force that attracts ions with high dielectric decrement to the interface.

Evidence for OH^−^ ion adsorption was demonstrated, in a simple pH-stat experiment emulsifying unreactive paraffin oils, that the surface charge density was measured at ~−5 to −6 µC cm^−2^ [11]. If this surface charge density is confined to a double layer just 1 nm thick, then this corresponds to a concentration of 0.5 M (a 5 nm thick double layer is 0.1 M in the surface charge species). What is surprising is the magnitude of that charge, and sceptics might reasonably question how such a large concentration of surface hydroxide had been overlooked for so long. The total surface charge is the quantity of base required to maintain a constant pH when hydroxide ions are adsorbed and hydronium ions are released; note that a little salt is needed to replace hydronium ions in the double layer. The droplet size is required to calculate the surface charge density. In our 2004 *Angewandte* paper, the electroacoustic technique was used, but other, older methods could have been employed [11]. The surface charge remains negative until the pH is less than about 3, i.e., in millimolar acid. This immediately led to the supposition that hydroxide ions adsorbed on the surface are responsible for the negative charge [3]. The charging observed at the air–water and solid-water interfaces due to hydroxide ion adsorption is responsible for many observed effects, as given below [5,31].

#### 1.1.1. Air–Water Interface

The surface charge density of the air–water interface has not been measured in this way because of the experimental difficulties of managing swarms of bubbles. The pH dependence of the zeta potential is so similar to that of the oil–water interface that it has been assumed they have similar properties. There are independent reasons to expect this similarity based on the explanation for the surface charge described below [31].

#### 1.1.2. Teflon–Water Interface

Confirmation of the large surface charge density at the aqueous–hydrophobe interface was provided by Lutzenkirchen from Karlsruhe in collaboration with the late Nikola Kallay [32]. They successfully measured the surface charge density of colloidal Teflon particles at −4 μC cm^−2^, in remarkable agreement with the value of −5 μC cm^−2^ at the oil–water interface. The presence of spontaneous charge on inert polymers accounts for electroosmotic effects, which had been attributed to impurities.

#### 1.1.3. Surface Tension

The surface tension of neat water should provide a measure of the ionic concentration at the interface. It is a paradox that the alleged interfacial hydroxide ions do not contribute to pH dependence of the surface tension. An explanation has been advanced that this is a consequence of the concentrations of hydroxide and hydronium ions being coupled by the ion product of water. In the absence of salt, protons must be adsorbed to maintain neutrality and the contribution of both ions cancels out. In pure water, the surface tension reflects adsorbed hydroxide ions. Hence, if the pH of a solution is decreased by one unit, the hydronium ion concentration increases by ten, but the hydroxide concentration also decreases by ten and the two effects cancel out.

This explanation does not apply to pH dependence in the presence of salt, where the pH is controlled by the addition of NaOH. For example, an increase in the surface tension arises between pH 4 and 7 only in the presence of added salt. This is consistent with a strong surface affinity of OH^−^ and not chloride or other anions [33]. Surface impurities would lead to a reduction in the surface tension. In the presence of salt, the cation acts as counterion to adsorbed hydroxide ions. Changes in pH will now alter the hydroxide ion activity and not affect the counterion activity, leading to pH dependence of the surface tension.

It is frequently but mistakenly assumed that the surface tension of water without added electrolyte is 73, but this is for the charged surface. This was dramatically demonstrated when the surface tension of a pristine drop was measured within a few microseconds of its creation, with a surface tension of ~90 mN m^−1^. It then relaxes to ~73. This confirms the expectation that the equilibrium surface tension of water of 73 mN m^−1^ is that of a charged surface [34].

#### 1.1.4. On-Water Catalysis

Acceleration of organic reaction rates when performed in aqueous media has been observed for some time [35]. It was shown that the reaction mixture must contain an aqueous phase and be heterogeneous, i.e., there must be an interface between the organic reactants and water. If a small amount of methanol was added to the aqueous phase, there was little difference in the rate of the reaction. However, the rate slowed considerably when enough methanol was used to make the reaction homogeneous. There was a significant solvent isotope effect, with a noticeably slower rate in D_2_O. The reactions that have been described as accelerated by the on-water effect are also known to be subject to acid catalysis [35,36]. The on-water effect can therefore be explained by a simple acid-catalysis mechanism facilitated by the strong adsorption of the hydroxide ion by-product at the oil–water interface. The fluctuation correlation model developed [37] and described below explains that the intrinsic charge that develops at the interface of water with low relative permittivity (low dielectric constant) materials occurs due to the stronger affinity of the hydroxide ion over other ions. Thus, in the presence of water, hydrophobic interfaces undergo acid-catalyzed reaction rates through the autolysis of water [38].

### 1.2. Fluctuation Mechanism

**Figure 2:**
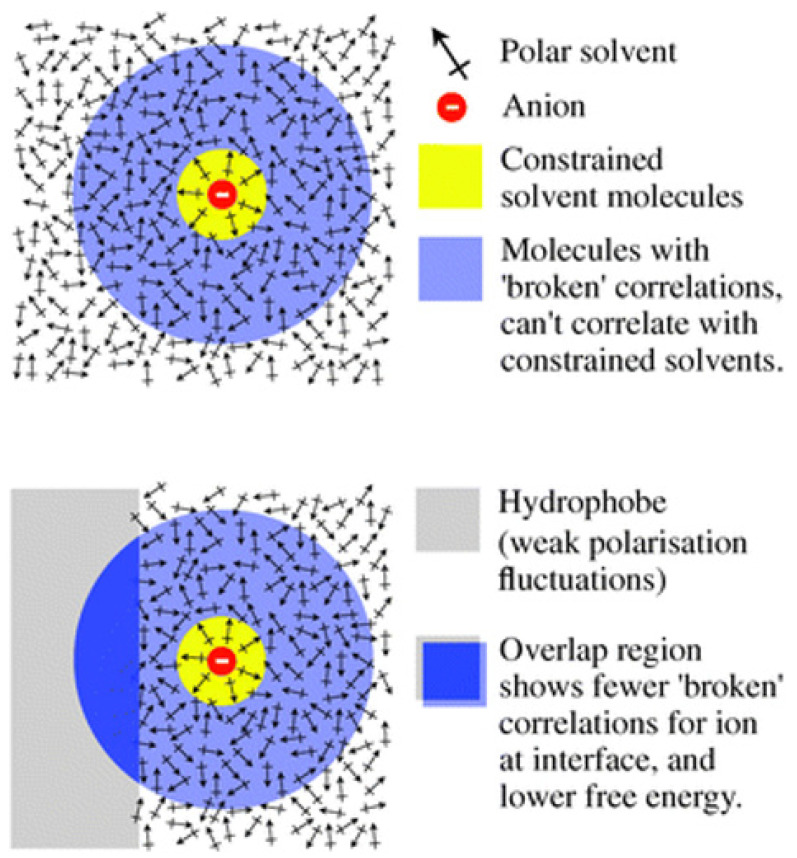
Concepts leading to surface affinity of hydroxide. (**Top**): An ion in a polar solvent is shown with a solvation shell of molecules that are not as free as the others to fluctuate (shaded inner circle represents the constrained solvation sphere). Molecules inside the larger shaded circle have their correlations with the ion’s hydration sphere ‘broken’. (**Bottom**): When the same ion is close to an interface with a hydrophobe, the free energy cost of the ion’s constrained environment is lower, because there are fewer solvent molecules whose correlations with the hydration sphere are broken by the ion (dark overlap of the correlation sphere with the hydrophobe).

Gray-Weale [37] recognized the requirement for a fundamental mechanism that operates in all three phases: solid, liquid and gas. He identified that the absence of molecular moment fluctuations of water near an ion exerts a force on the ion that attracts it to regions where the density of dipole-moment fluctuations is lower than in bulk water, i.e., to regions of lower relative permittivity (Figure 2). The force that drives the hydroxide ions to the surface arises from their suppression of correlation with the collective dipole moment fluctuations of surrounding water molecules. In principle, any ion would feel this force. It is especially strong for the hydroxide, with a binding free energy shown to be around 16–20 k_B_T, because the hydroxide combines a large dielectric decrement with a small associated region of reduced dipolar fluctuations. This estimate of k_B_T is similar to that obtained directly from the experimental isoelectric point. The isoelectric points described above for oils, air and inert polymers are in the range pH 2–4. At these pHs, the acid concentration, [H^+^], is 10^12^–10^8^ greater than the hydroxide ion concentration, [OH^−^], yet in the double layer the isoelectric condition requires the concentrations of the two species to be the same. Hence the preferential adsorption of the hydroxide ions generates a large equilibrium constant in favor of hydroxide over hydronium ions at the interface.

Recently, an experimental value for the energy of binding of hydroxide ions to the hydrophobic surface of hexadecane was reported. Gan and colleagues used second-harmonic generation spectroscopy to observe that hydroxide ion adsorption is almost saturated at 1 mM, consistent with zeta potential observations, with an adsorption free energy of −8.3 kcal mol^−1^, equivalent to 14 k_B_T [17]. They observe that, at high pH, when the interface is saturated with hydroxide, further addition of NaOH produced a similar effect to the addition of NaCl, i.e., there was no further special effect of hydroxide. This accounts for the claim that a pH indicator used to measure the water surface pH showed the surface pH to be 1.7 units lower than the bulk [39]. The pK_a_ of the indicator was 11.4; the measurements are not relevant to the question of the pH of the interface at pH ~5.

## 2. Results

### 2.1. Thermochemistry of Pristine Emulsions

Oil-in-water (O/W) emulsions are unstable, yet ‘surfactant-free’ O/W emulsions exist, in which water itself acts as the stabilizer. Autolysis of water creates an electrostatic stabilization of the emulsion as the O/W interface becomes charged by the adsorption of hydroxide ions. When such emulsions are broken by the addition of acid, there are only three significant contributions to the heat of reaction: the desorption (Heat_D_) and then neutralization (Heat_N_) of the surface hydroxide ions and the release of the interfacial tension (Heat_T_). Two of these terms can be assessed independently: the heat of neutralization of the hydroxide ions (Heat_N_) is obtained from the quantity of the adsorbed hydroxide ions measured in the emulsification process, or the surface charge density times the surface area, together with the well-known reaction heat of a strong acid with a strong base (−56 kJ mol^−1^). The small background heat of neutralization of acid and base at pH 9 (0.56 J) was subtracted from the measured experimental heats. The interfacial tension term (Heat_T_) is calculated from the measured size of the emulsion drops and the interfacial enthalpy of the oil (γ-Tdγ/dT). The heat of desorption of the hydroxide ions from the oil–water interface (Heat_D_) is not directly accessible, but it can be obtained from the difference between the overall measured heat of reaction and the sum of the two terms described above. This experimental value can then be compared to the theoretical estimate made from the fluctuation–correlation model of the hydrophobic force. Hence, the measured heat provides a direct test of the allegedly controversial role of the hydroxide ion in stabilizing the O/W emulsions and a quantitative description of the hydrophobic effect. 

**Scheme 1:**
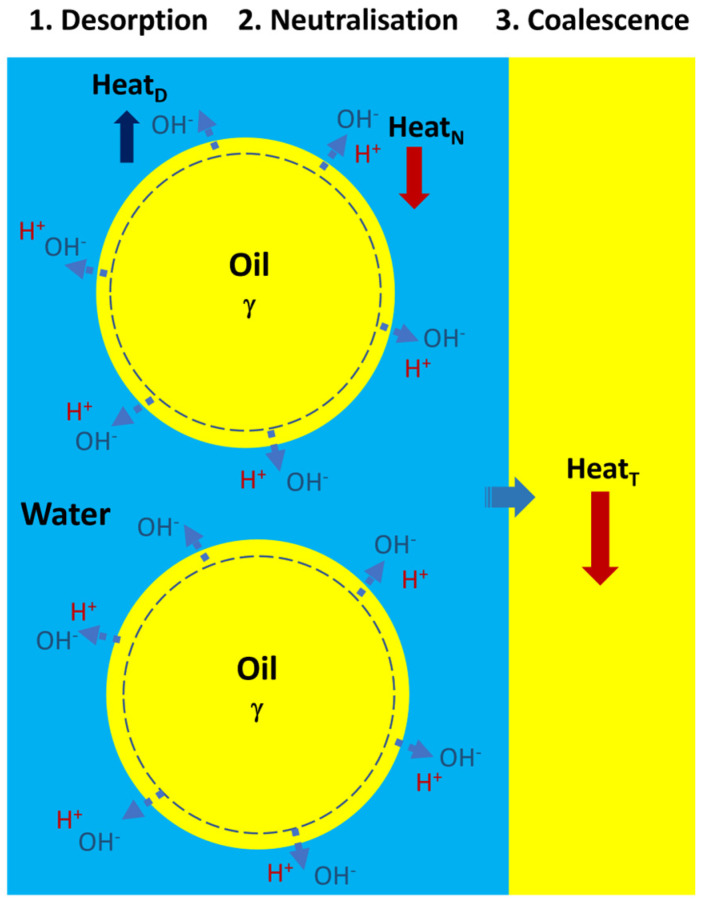
Coalescence of hydroxide ion stabilized emulsion droplets and the release of heat.

The heat released when the emulsion is broken by neutralization of the hydroxide electrostatic stabilization is given as Heat_exp_. This is the experimentally measured heat less 0.56 J L^−1^ to account for the heat of neutralization from pH 9 to pH 7 of the 10^−5^ M background NaOH electrolyte. Further dilution of the resultant 10^−5^ M NaCl solution has negligible heat. The heat of neutralization of the hydroxide ions if they were free in solution is shown as Heat_N_. This is calculated from an average surface charge of −5.5 μC cm^−2^, the surface area and the heat of neutralization of −56 kJ mol^−1^. The heat released when the uncharged emulsion drops coalesce (Heat_T_) is given by the surface area times the interfacial enthalpy (Scheme 1). The difference between the experimental heat and the sum of these two terms is the heat required to desorb the hydroxide ions, or, in the opposite direction, the heat released when the hydroxide ions adsorb at the oil–water interface (Heat_A_).

**Figure 3:**
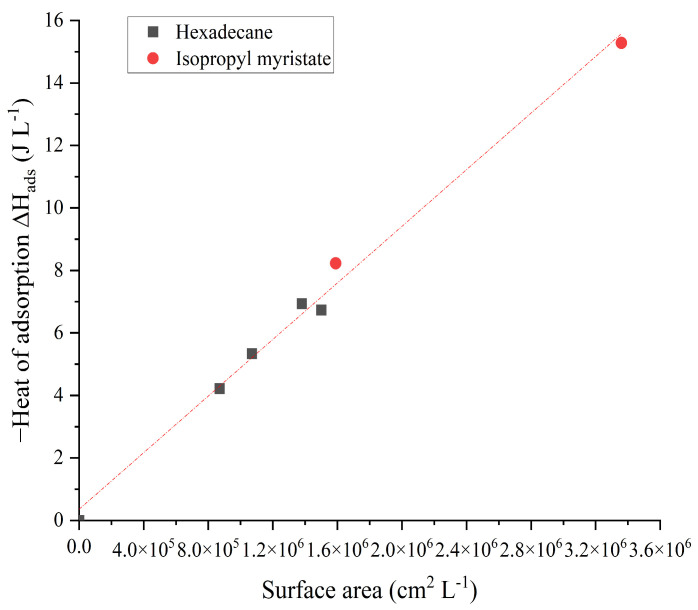
Heats of adsorption (∆H_A_) of both hexadecane (■) and isopropyl myristate (●) emulsions as a function of interfacial area. R^2^ = 0.990.

To test the validity of this model emulsions were also prepared with isopropyl myristate in place of hexadecane. This oil has a very low water solubility, like hexadecane, but with a much lower interfacial tension, about half that of hexadecane. The consequence is that it forms emulsions much more readily, attaining droplets of about half the size of the smallest hexadecane sample. The inclusion of the two isopropyl myristate emulsions along with the hexadecane emulsions are plotted in Figure 3 as a function of the emulsion surface areas. The fact that the two different oils are in the same linear relationship indicates that there are no major terms missing from the analysis. The slope gives the heat of adsorption per unit area, 4.38 ± 0.11 × 10^−6^ J L^−1^ cm^−2^ or 4.38 × 10^−20^ J L^−1^ nm^−2^. From the surface charge density, it can be calculated that each hydroxide occupies about 2.7 nm^2^. The heat of adsorption of each hydroxide ion is thus 1.17 × 10 ^−19^ J, or 28.4 k_B_T at 298 K. This is in very good agreement with the theoretical prediction of ~20 k_B_T. The fluctuation correlation model describes the force driving ions with high dielectric decrement to regions of low dielectric permittivity and assumes that the reduction in fluctuations is concentrated at the ion’s site, which underestimates the strength of this force. The region of reduced dipolar fluctuations is also unchanged until it touches the interface and the potential is valid at distances greater than the solvated ion’s size but, at distances less than this, no force acts on the ion. The neglecting of higher order terms for the potential also underestimates this force. When the hydroxide ion’s solvation sphere reaches the surface, the mechanism is no longer important and other effects take over, such as image charge repulsion, which is accounted for, and ion repulsion due to electronic dipole fluctuations. The latter is ignored to maintain simplicity. The force attracting ions to the interface also ignores other ions, such as chloride and hydronium ions, as their potential well is much shallower than hydroxide’s.

### 2.2. Adsorption of Alkylammonium Salts

The role of hydroxide ions could be considered as special, but the fluctuation correlation model predicts that other ions with a high dielectric decrement should also be surface active. One such is the tetra-*n*-butylammonium cation [Bu_4_N]^+^. The dielectric decrement of [Bu_4_N]Br is 26.0 M^−1^ [40], which is much larger than the value of 20.9 M^−1^ for NaOH [41]. It is known that the tetraalkylammonium ions reduce the surface tension of water with increasing effect as the alkyl chain length increases [42]. Consequently, the dipole correlation force model predicts that [Bu_4_N]^+^ cation should stabilise positively charged oil-in-water emulsions, just as the hydroxide ion stabilizes negatively charged ones. In contrast, the tetraethylammonium cation [Et_4_N]^+^ with a dielectric decrement of 17.3 would be expected to compete with the OH^−^ ion under slightly acidic to basic conditions. We tested the tetra-*n*-butylammonium cation [Bu_4_N]^+^ and tetraethylammonium cation [Et_4_N]^+^. The dielectric decrement of [Bu_4_N]Br is 26.0 M^−1^ [40], and that of [Et_4_N]Cl is 17.3. Compared to the value of 20.9 M^−1^ for NaOH, it would be expected that [Et_4_N]^+^ would compete with OH^−^ for surface activity.

**Figure 4:**
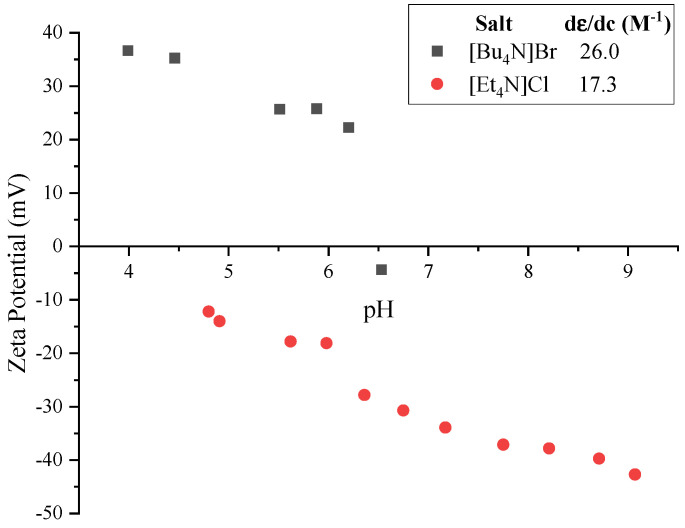
Zeta potential as a function of pH for 2 vol % hexadecane emulsions stabilized with 0.2 × 10^−3^ M TBAB at pH 4, or 0.15 × 10^−3^ M TEAC at pH 9.0.

**Figure 5:**
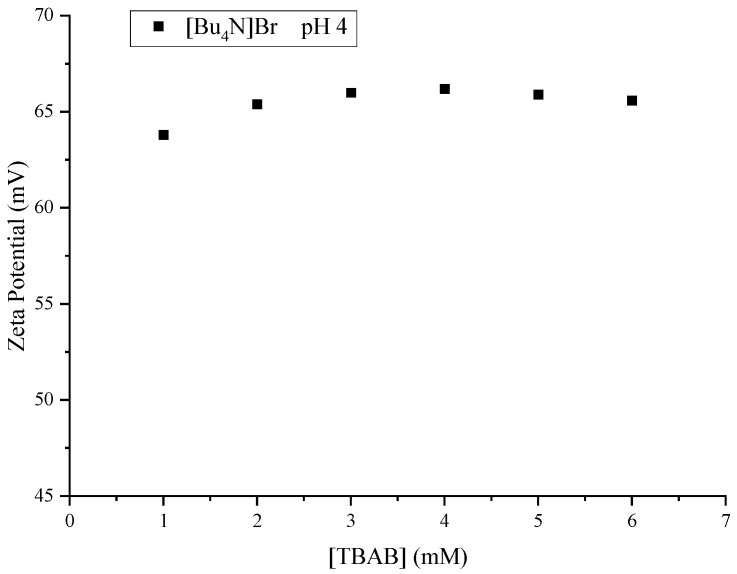
Zeta potential as a function of tetrabutylammonium bromide (TBAB) concentration for a 2 vol % hexadecane emulsion stabilized with 1.0 × 10^−3^ M TBAB at pH 4.

The mobility and resulting zeta potential of hexadecane emulsion drops as a function of alkylammonium salt, pH and concentration, is shown in Table 1 and Table 2. A stable emulsion with a positive zeta potential of +41 mV was prepared when hexadecane was emulsified in 0.2 mM [Bu_4_N]Br at the natural pH of water of ~6.0. This is in sharp contrast to the negative zeta potential of −50 mV observed at this pH for emulsions formed in the absence of [Bu_4_N]^+^, charged by the adsorption of hydroxide ions [43]. When more [Bu_4_N]Br is added up to 4 mM, the positive zeta potential increases to +52 mV (Table 1). If the initial pH of the 0.2 mM [Bu_4_N]Br solution is reduced to pH 4 with the addition of HBr and then emulsified with hexadecane, a similarly positively charged emulsion is produced with a zeta potential of +37 mV. Increasing the pH by addition of [Bu_4_N]OH solution resulted in a gradual decrease in the zeta potential to +22 mV at pH 6.2, followed by a sharp decrease to an unstable emulsion with a small negative zeta potential at pH 6.5 (Figure 4). Thus, the isoelectric point of this emulsion is pH 6–7, again in contrast to an isoelectric point of pH 2–3 for the hydroxide stabilized emulsions. Attempts to prepare emulsions in 0.15 mM [Bu_4_N]OH between pH 7 and 9 with the pH adjusted with HBr were unsuccessful, presumably because of competition between the positive [Bu_4_N]^+^ and negative OH^−^. At pH 4, the surface of the emulsion appears to be almost saturated at 1 mM [Bu_4_N]Br. Emulsification with this initial concentration produced an emulsion with a zeta potential of +64 mV. Addition of more [Bu_4_N]Br to 3 mM increased the zeta potential very slightly to +66 mV, with no further increase up to 6 mM (Figure 5).

**Scheme 2:**
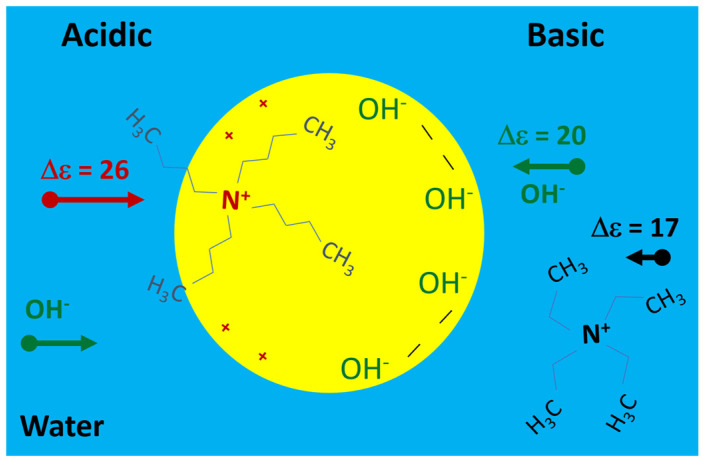
Adsorption of hydroxide or tetrabutylammonium ions at the hydrophobic oil–water interface. Ions with high dielectric decrements show stronger surface activity.

To test the effect of alkyl chain length on the adsorption, TEAC was added and the pH raised to 9 with the addition of NaOH. The emulsification was maintained at pH 9 with NaOH, and then measurements were recorded from pH 9 to 4.8 with HCl used to lower the pH (Figure 4). The emulsion was prepared with 0.2 mM tetraethylammonium chloride, [Et_4_N]Cl, instead of the tetrabutylammonium salt. The dielectric decrement of [Et_4_N]Cl is only 17.3 [40], so it is not expected to compete successfully with hydroxide ions in giving positively charged droplets. This was found to be the case. At pH 9 the emulsion had a zeta potential of −43 mV. Addition of HCl gradually reduced the negative zeta potential to −12 mV at pH 4.8, but it never became positive. Over the entire pH range, the surface of the drops remained negative and approached zero below pH 4.8, consistent with NaOH-stabilized emulsion drops in the absence of alkylammonium salts (Figure 4, Scheme 2).

## 3. Discussion

### 3.1. Importance of the Hydroxide Ion

An explanation has recently been advanced for the ubiquitous adsorption of hydroxide ions at interfaces of water with low dielectric surfaces [37]. The force that drives the hydroxide ions to the surface arises from their suppression of correlation with the collective dipole moment fluctuations of surrounding water molecules. The extent of this can be minimized if the ion approaches a medium of low dielectric constant, thereby reducing the number of water molecules affected. The strength of this affinity therefore depends on the dielectric decrement and on its effective hydration radius, which may not be related to the ionic radius [44]. All electrolytes reduce the dielectric constant of their solutions in water, but hydroxide ions have a particularly large dielectric decrement. This, combined with its small size, leads to its stronger adsorption than for most other monovalent ions, and particularly more than for the hydronium ions. Interfaces considered hydrophobic can therefore acquire a negative charge through the autolysis of water and the strong affinity of hydroxide ions to low dielectric surfaces. Hydroxide ion adsorption explains the rate enhancements of organic reactions through acid catalysis in proportion to the interfacial area generated via homogenization or rapid stirring.

There are few modern values for the dielectric decrements of strong acids. From dielectric relaxation data from Lileev [45], the determined value for HCl is ~24 M^−1^. This value is reasonably high compared with an NaOH value of 20 and would seem to require strong adsorption of hydronium ions instead of hydroxide. A second factor in the Gray–Weale tension model is the distance of closest approach of the ion to the interface. It is known that hydronium ions are quite large, involving 6–7 water molecules [46,47]. We speculate that this could inhibit the adsorption. The suppression of fluctuations by the hydroxide is consistent with what we know of its unusual solvation structure from other sources. Four water molecules are tightly coordinated to the hydroxide’s oxygen. It is plausible that hydroxide’s high dielectric decrement is related to this structure, perhaps because the four waters are so tightly bound, and the square geometry can only awkwardly be accommodated by the surrounding random tetrahedral network. The four water molecules bound to the hydroxide, along with others in a second solvation shell, are unable to respond freely to an electric field, and so the presence of the hydroxide ion lowers the relative permittivity. The effect is not seen for other singly charged monatomic ions.

Large and polarizable ions are known to be attracted to air interfaces where they are enriched. The attraction was shown to increase exponentially with ion radius [48] but is not necessarily correlated to polarizability [49]. However, the electrospray experiments were made on surfaces formed by a liquid microjet with a lifetime of less than a millisecond. As a result, the surface would unlikely be sufficiently charged by the adsorption of hydroxide ions, since the autolysis of water is slow, the maximum value of the dissociation rate constant, k_d_, being 10^−3^ s^−1^ [44]. Surface affinities of halide ions are also not as dominant as hydroxide ion adsorption and, as a result, zeta potential measurements and surface spectroscopic measurements conducted on equilibrated surfaces show a weak halide ion dependence. Both the effective hydration radius and dielectric decrement are important for the surface affinity at the hydrophobic–water interface. Although halide ion surface affinities increase in the order I < Br < Cl < F [50], which is the opposite order to the dielectric decrement of these ions [51], one would expect fluoride to be more weakly adsorbed due to its smaller dielectric decrement. The difference is attributed to ion pairing of small fluoride and sodium counterions that are condensed with the high surface hydroxide ion concentration [44].

### 3.2. Hydrophobic Ions

The force driving ions to hydrophobic surfaces is not restricted to the hydroxide ions. Ions with larger dielectric decrements would compete with hydroxide ions generated from the autolysis of water. In mM solutions of [Bu_4_N]Br between pH 4 and 6, hexadecane can be emulsified to produce positively charged droplets. This is in sharp contrast to the negatively charged droplets that occur at this pH in the absence of alkylammonium salt. At the iep of about pH 6.3 to 6.5, the interface presumably contains equal numbers of positive [Bu_4_N]^+^ cations and negative OH^−^ anions. As this occurs with a [Bu_4_N]Br concentration of 2 × 10^−4^ M and a hydroxide ion concentration of 2–3 × 10^−8^ M, it implies that the hydroxide ion is adsorbed about 10^4^ times more strongly than [Bu_4_N]^+^. This occurs despite the larger dielectric decrement of [Bu_4_N]Br because the cation is also much larger than the hydroxide anion. The radius of [Bu_4_N]^+^ is reported to be 4.13 Å [52]. Hence, it cannot approach the interface nearly as closely as can the hydroxide ion, and thus cannot reduce as effectively the number of water molecules affected by the suppression of the dipole correlation [37]. The critical parameter is not the ion size but the size of the region where polarization fluctuations are reduced below the bulk value. Polyatomic monoatomic ions, like PF_6_^−^ ions, have been shown to have a strong surface enhancement [48]. The fluctuation correlation model predicts that such ions, including TBAB, will also be attracted by the same mechanism, not because they constrain water molecules but because each ion is a region from which water molecules are absent. Therefore, TBAB, with a high dielectric decrement, should also show such surface affinity, whereas TEAC does not do this as effectively due to its lower dielectric decrement compared to hydroxide ions and must therefore compete with the surface.

Winter et al. have measured the adsorption of [Bu_4_N]I at the air–water interface by photoelectron spectroscopy of a water microjet [53]. They observe a monolayer coverage at about 0.024 molal concentration, well in excess of the 1 mM saturation concentration described above from the zeta potential measurements on the hexadecane–water interface. It may be that, as the surface charge increases further, adsorption of both cations and counter-anions occurs inside of the shear plane, without any further increase in the zeta potential. Indeed, their analysis assumes the presence of ion pairs at the surface.

The fluctuation correlation model has been demonstrated for hydroxide ions and tetra-butyl ammonium salts. Comparison with other methods and theories relating to spectroscopic methods do not, in general, show an excess of hydroxide ions at the interface. This is most likely to be because hydroxide ions are below the outermost molecular layers. The adsorption of other anions is not a contradiction to our work. Petersen and Sakally [54] indicate that spectroscopic methods do not detect an excess of hydroxide between pH 9 and 14. They suggest that the two outermost water layers dominate the signal and, just because an ion exhibits surface enhancement via spectroscopy, this does not imply that it is enhanced in the entire interfacial region to exhibit a net surface excess. Spectroscopy probes the top couple of water layers, whereas the zeta potential is typically determined at the shear plane at depths of 2–3 nm. There is, therefore, a need to distinguish between the different definitions of the ‘interface’ for each experiment.

Simulation studies of hydronium ions show that they do not strongly distort the hydrogen bonding network [55,56]. This suggests that the hydration sphere of hydronium is not as tightly bound as it is for hydroxide. The tightly constrained hydration sphere is responsible for the high dielectric decrement of hydroxide. Hydroxide ions are not seen in classical MD simulations of ion adsorption due to the small system size, and to values of hydroxide dielectric decrement lower than the actual ion. MD simulations of electric fields applied to oil droplets in water show that the drops experience a force due to the ordering of dipolar water molecules at the interface. We agree that this occurs, but neglect dipolar ordering to maintain simplicity. We also do not include hydronium ion binding due to the binding energy being much weaker than that of hydroxide. Ultimately, a more comprehensive theory should include all sources of charge at hydrophobic–water interfaces.

The current understanding of how hydrophobic–water interfaces are charged and stabilized should ultimately be included in models and theories that assume neutral hydrophobic surfaces. The stability of such interfaces can be controlled by optimizing conditions to enhance or reduce adsorption of ions that would otherwise not be expected to adsorb. We demonstrated that this charge is substantial, being −5 to −6 µC cm^−2^ for OH^−^. In pure water-based systems, at neutral pH hydroxide ions still adsorb, although at a lower charge density, and even in the presence of conventional surfactants such as SDS. At higher pH, this effect is stronger.

The observation that both the adsorption of hydroxide ion and the adsorption of the tetrabutylammonium cation can be explained by the force arising from suppression of water dipole correlations has profound implications. One is that the hydroxide adsorption is not unique and hence is not the consequence of the effect of the surface on the autolysis constant of water [57]. The autolysis of water is enhanced at the interface, but this is a consequence of the force that causes the strong adsorption of the hydroxide ion. The [Bu_4_N]^+^ cation is generally regarded as hydrophobic [58], so a simple interpretation of its attraction to the hexadecane or to the air interface invokes a hydrophobic force. However, the hydroxide ion is not considered hydrophobic. While separate explanations are possible for the two different cases, the availability of a single comprehensive explanation is attractive. It has not escaped our notice that the force we have postulated arising from the suppression of water dipole moment fluctuations immediately suggests a possible alternative explanation of hydrophobic effects [59].

## 4. Materials and Methods

Oil-in-water emulsions were prepared with a piston homogenizer at a concentration of 2 vol% oil in 0.2 mM NaCl at pH 9 [60]. The pH was maintained with 25 mM NaOH. The zeta potential of the emulsion was determined from the mobility measured by the electroacoustic technique with a ZetaProbe instrument (Colloidal Dynamics LLC, North Attleboro, MA, USA). The size of the emulsion drops was measured by acoustic attenuation, which depends on both thermal conduction and scattering losses. The measured attenuation was fitted to the ultrasonic scattering data given in Table 3. Tetraethylammonium chloride (TEAC), tetrabutylammonium bromide (TBAB), tetrabutylammonium hydroxide (TBAOH), NaOH, HCl and HBr were used as received. Hexadecane (Sigma, St. Louis, MO, USA, 99+%) and isopropyl myristate (Sigma 99+%) were purified by passing through a basic alumina column five times.

The emulsion at pH 9 was broken by addition of acid to about pH 3. The surface hydroxide ions are desorbed and then neutralized by acid and the now uncharged emulsion drops coalesce with the release of the interfacial tension energy. The heat released is measured by the microcalorimeter (MicroCal iTC 200) and converted to J L^−1^ for the 2 vol% emulsion. The results are presented in Table 3, where different sized emulsions of hexadecane or isopropyl myristate were obtained by emulsifying at different times during the homogenization process. The surface areas were calculated from the polydisperse equation [11]: SA = SA_o_ e^0.5 ln2 (d85/d15)^, where SA_o_ is the surface area of a monodispersed emulsion.

For the adsorption of alkylammonium salts, TBAB (0.2 mM) was added to the water phase and emulsified either at pH 4 with the addition of HBr, or pH 9 with the addition of TBAOH. After about 2 h of homogenization, the mobility of the drops was measured at pH 9 or 4. The sample prepared at pH 9 was less stable so no further additions could be made. The sample prepared at pH 4 was more stable and, after the initial mobility was measured, more TBAB was added (up to ~4 mM) and the mobility recorded. Another sample was pH titrated from pH 4 to ~pH 7 with the addition of TBAOH to determine the isoelectric point of the emulsion. Emulsions made at pH 9 using only TBAOH (~0.1 mM), did not form properly. TEAC was used as a comparison and homogenised at pH 9 with the addition of NaOH to raise the pH. The mobility was recorded after the emulsion formed, and after each addition of HCl, to lower the pH to 4.80.

## 5. Conclusions

In summary, two independent tests of the fluctuation correlation model have been performed. The first was that the predicted binding energy of the hydroxide ion at the hydrophobic–water interface, estimated to be ~16–20 k_B_T, was correct. Our measured value of 28 k_B_T, based on determining the heats evolved during neutralisation and coalescence of hexadecane and isopropyl myristate emulsions, is in good theoretical agreement. The second test demonstrated that hydroxide ions are not special and that hydrophobic ions with dielectric decrements larger than that of the hydroxide ion should preferentially adsorb at hydrophobic surfaces, so long as their distance of close approach is not hindered by their solvation. We demonstrated this with TBAB, giving positive zeta potentials below pH 6. In contrast, TEAC with a smaller dielectric decrement did not preferentially adsorb. No present theory can account for both positive and negative interfaces with the demonstrated pH dependence. Such ions are able to reduce water dipolar fluctuations and the dielectric constant of interfacial water, causing a Hamaker-like force that drives the ions to the hydrophobic interface, where the dipole moment fluctuations are lower than in the bulk water.

## Figures and Tables

**Table 1:** Mobility and zeta potential of 2 vol % hexadecane emulsions stabilized with tetrabutylammonium bromide salt, TBAB, below pH 7.

System	[Bu_4_N]Br (mM)	pH	K (S m^−1^)	μ (10^−8^ m^2^ V^−1^ s^−1^)	ζ (mV)
**TBAB**	0.20	6.00	0.0052	+2.54	+41
	0.40		0.0075	+2.79	+46
	0.60		0.0096	+2.89	+48
	1.00		0.0138	+3.01	+49
	4.00		0.0563	+3.38	+52
**TBAB/TBAOH**	0.20	3.99	0.0069	+2.24	+36.7
		4.46	0.0064	+2.12	+35.3
		5.51	0.0067	+1.20	+25.7
		5.88	0.0068	+0.92	+25.8
		6.20	0.0092	+0.29	+22.3
		6.53	0.0145	−0.16	−4.3
**TBAB**	1.00	4.13	0.0161	+4.00	+63.8
	2.00	3.98	0.0272	+4.23	+65.4
	3.00	4.02	0.0372	+4.23	+66.0
	4.00	4.05	0.0467	+4.30	+66.2
	5.00	4.06	0.0558	+4.26	+65.9
	6.00	4.07	0.0645	+4.19	+65.6

**Table 2:** Mobility and zeta potential of 2 vol % hexadecane emulsions stabilized with 0.2 × 10^−3^ M tetraethylammonium chloride, TEAC, as a function of pH.

System	pH	K (S m^−1^)	μ (10^−8^ m^2^ V^−1^ s^−1^)	ζ (mV)
**TEAC/HCl**	9.07	0.0137	−2.77	−42.7
	8.71	0.0133	−2.69	−39.7
	8.21	0.0131	−2.51	−37.8
	7.75	0.0130	−2.42	−37.1
	7.17	0.0131	−2.27	−33.9
	6.75	0.0133	−2.06	−30.7
	6.36	0.0135	−1.86	−27.8
	5.98	0.0141	−1.19	−18.1
	5.62	0.0142	−0.98	−17.8
	4.91	0.0150	−0.49	−14.0
	4.80	0.0149	−0.42	−12.2

**Table 3:** Experimental heats of adsorption (Heat_exp_) and calculated heats (Heat_N_, Heat_T_ and Heat_A_) of both hexadecane and isopropyl myristate emulsions as a function of total interfacial area.

Oil	d (nm)	d_15_–d_85_ (nm)	Surface Area (cm^2^ L^−1^)	−Heat_exp_ * (J L^−1^)	−Heat_N_ (J L^−1^)	−Heat_T_ (J L^−1^)	+Heat_A_ (J L^−1^)
**Hexadecane**	2000	1300–3077	8.7 × 10^5^	5.21	2.76	6.67	−4.22
	1331	991–1789	1.07 × 10^6^	6.26	3.40	8.20	−5.34
	1345	844–2144	1.38 × 10^6^	8.02	4.38	10.58	−6.94
	940	706–1250	1.50 × 10^6^	9.52	4.76	11.50	−6.73
**IPM**	760	722–800	1.59 × 10^6^	4.04	5.05	7.23	−8.23
	580	355–949	3.36 × 10^6^	10.65	10.66	15.27	−15.28

* 0.56 J was subtracted from experimental heat to account for background neutralisation from pH 9 to pH 7. Acoustic attenuation parameters for hexadecane with values for isopropyl myristate (IPM) shown in parentheses at 25 °C. Density = 0.77 g cm^−3^ (0.85); heat capacity = 2214.5 J kg^−1^ K^−1^ (2019); speed of sound = 1338 m s^−1^ (1326); thermal conductivity = 0.143 W m^−1^ K^−1^ (0.143); thermal expansion coefficient = 8.98 × 10^−4^ K^−1^ (approximated as 8.00 × 10^−4^); intrinsic attenuation = 10 × 10^−14^ Np m^−1^ s^2^ (10 × 10^−14^); interfacial tension = 52.5 ± 0.4 mN m^−1^ (21.3 ± 1.3). The surface charge density (−5.5 ± 0.6 μC cm^−2^), surface areas, and experimental heats were used assuming a 10% error in the determined or measured values. Values for dγ/dT = −0.081 were used for hexadecane and isopropyl myristate.

## Data Availability

The original contributions presented in this study are included in the article. Further inquiries can be directed to the corresponding authors.
